# Use of Evidence-Based Best Practices and Behavior Change Techniques in Breast Cancer Apps: Systematic Analysis

**DOI:** 10.2196/14082

**Published:** 2020-01-24

**Authors:** Kerstin Kalke, Tamar Ginossar, Joshua M Bentley, Hannah Carver, Sayyed Fawad Ali Shah, Anita Y Kinney

**Affiliations:** 1 Department of Communication Studies Northwestern University Evanston, IL United States; 2 Department of Communication and Journalism University of New Mexico Albuquerque, NM United States; 3 Department of Strategic Communication Texas Christian University Fort Worth, TX United States; 4 Milken Institute School of Public Health George Washington University Washington, DC United States; 5 Department of Communication Jacksonville State University Jacksonville, AL United States; 6 Department of Biostatistics and Epidemiology School of Public Health Rutgers University New Brunswick, NJ United States; 7 Cancer Institute of New Jersey Rutgers University New Brunswick, NJ United States

**Keywords:** mHealth, breast cancer, mobile apps, health behavior, health apps

## Abstract

**Background:**

Theoretically designed mobile health (mHealth) breast cancer interventions are essential for achieving positive behavior change. In the case of breast cancer, they can improve the health outcomes of millions of women by increasing prevention and care efforts. However, little is known about the theoretical underpinnings of breast cancer apps available to the general public.

**Objective:**

Given that theories may strengthen mHealth interventions, this study aimed to identify breast cancer apps designed to support behavior change, to assess the extent to which they address content along the cancer care continuum and contain behavior change techniques, and to assess the degree to which star rating is related to theory-based design.

**Methods:**

Using a criteria-based screening process, we searched 2 major app stores for breast cancer apps designed to promote behavior change. Apps were coded for content along the cancer care continuum and analyzed for behavior change techniques. The Mann-Whitney *U* test was used to examine the relationship between star ratings and the use of behavior change techniques in apps with star ratings compared to those without ratings.

**Results:**

The search resulted in a total of 302 apps, of which 133 were identified as containing breast cancer content. Only 9.9% (30/302) of apps supported behavior change and were further analyzed. These apps were disproportionally focused on behaviors to enhance early detection, whereas only a few apps supported care management, treatment, and posttreatment behaviors. Regarding theories, 63% (19/30) of apps customized content to users, 70% (21/30) established a health-behavior link, and 80% (24/30) provided behavior change instructions. Of the 30 apps, 15 (50%) prompted intention formation whereas less than half of the apps included goal setting (9/30, 30%) and goal reviewing (7/30, 23%). Most apps did not provide information on peer behavior (7/30, 23%) or allow for social comparison (6/30, 20%). None of the apps mobilized social norms. Only half of the apps (15/30, 50%) were user rated. The results of the Mann-Whitney *U* test showed that apps with star ratings contained significantly more behavior change techniques (median 6.00) than apps without ratings. The analysis of behavior change techniques used in apps revealed their shortcomings in the use of goal setting and social influence features.

**Conclusions:**

Our findings indicate that commercially available breast cancer apps have not yet fully realized their potential to promote behavior change, with only a minority of apps focusing on behavior change, and even fewer including theoretical design to support behavior change along the cancer care continuum. These shortcomings are likely limiting the effectiveness of apps and their ability to improve public health. More attention needs to be paid to the involvement of professionals in app development and adherence to theories and best practices in app design to support individuals along the cancer care continuum.

## Introduction

### Background

Globally, more than 1 million women are diagnosed with breast cancer every year, making it the most common cancer type among women [1]. With an estimated 410,000 individuals dying from the disease annually, breast cancer constitutes the leading cause of death from cancer among women across the globe [1,2]. Breast cancer morbidity and mortality can be reduced through the promotion of exercise, healthy diet, and limited alcohol intake and adequate access to screening services, treatment, and care management. However, many women lack information and support for behavior change along the cancer care continuum [2-4]. The cancer care continuum provides a framework to evaluate care plans, priorities, progress, and research gaps, as it refers to the various stages of cancer etiology, prevention, early detection, diagnosis, treatment, survivorship, and end-of-life care [5]. For example, advances in screening and treatment help increase the number of breast cancer survivors. In the United States alone, 3.5 million women are breast cancer survivors [6]. They have specific social, psychoemotional, health care, diet, and exercise needs that are different from the general public, and interventions should target these specific needs [7].

Mobile phone apps are promising platforms to extend current health care efforts and to reduce health disparities. Mobile phone ownership is growing rapidly across countries, and health and medical apps are becoming increasingly popular [8]. A national survey on the use of health apps among mobile phone owners in the United States showed that more than half of all mobile phone users have downloaded health-related apps and, of those, two-thirds felt that health apps improved their health [9].

The use of mobile communication technologies for health purposes (mobile health, mHealth) shows great promise in supporting health-related behavior change [10]. Apps have been used in a variety of health contexts with a wide spectrum of functions, ranging from supporting weight loss and physical exercises to the management of chronic diseases [11]. Research on the effectiveness of mHealth interventions strongly supports the integration of behavior change theories into app content and design, and guidelines have been developed and validated to measure the degree to which apps use theoretically based design, such as the taxonomy of behavior change techniques [10,12]. A systematic review of mHealth interventions showed that apps that were based on theoretical constructs significantly increased behavior change efforts [10]. Of the studies included in the review, the majority of interventions used action and feedback cues and social support as theoretical constructs, whereas the most prominent theories included the social cognitive and self-determination theories. Other studies reported success in pain assessment and management through mobile apps by using diaries and direct provider feedback [11].

In the context of breast cancer, apps have the potential to support healthy behaviors along the cancer care continuum, and studies have explored the potential of apps in the prevention, treatment, and management of breast cancer. The following sections provide an overview of how apps have been used to support behavior change and disease management on the cancer care continuum, from prevention and risk to diagnosis, treatment, survivorship, and end-of-life care.

#### Prevention, Risk Assessment, and Screening

mHealth interventions targeting cancer preventive behaviors have shown some success in encouraging preventive behaviors through the integration of behavioral constructs in intervention design, such as text message reminders, tailored feedback, and narratives [13]. Primary preventive behaviors include being physically active, maintaining a healthy weight, reducing the use of tobacco, limiting alcohol intake, and eating a healthy diet. Apps have been used to promote these behaviors by providing information about risk reduction strategies and by offering tracking features to monitor dietary intake and physical exercise [14].

Furthermore, breast cancer screenings can help detect the disease early on, thus increasing treatment options and chances of survival [15]. Mammograms are the only screening method that has shown to increase longevity [15-17]. Certain women who have a family history of breast or ovarian cancer or are of Ashkenazi Jewish heritage are further advised to undergo genetic testing for BRCA1 or BRCA2 mutations and may benefit from regular and enhanced screenings at an earlier age [15]. Apps have been used to assist in the promotion of regular screening for breast cancer and have shown to increase knowledge of breast cancer screening guidelines as well as screening attendance [18,19]. However, there is a considerable body of evidence indicating that increasing knowledge alone has limited to no effectiveness in changing health behavior [20-22].

#### Diagnosis, Management, and End-of-Life Care or Survivorship

Apps can provide targeted and personalized information to patients with breast cancer after diagnosis and during and after treatment. Diagnosis-related information includes cancer stage, tumor type, and prognosis. For patients and survivors, mHealth interventions have been used to facilitate disease management, support patient-provider communication, increase patients’ quality of life, and enhance self-care strategies [23,24]. For example, Uhm et al [25] had success in increasing physical activity in breast cancer patients through the use of an app coupled with a pedometer. An mHealth-supported behavioral counseling intervention resulted in positive physiological changes, including weight loss and increased vegetable and fruit intake [26]. In a qualitative assessment of an app supporting patients with breast cancer during treatment, patients reported that they found audio recordings of conversations and personalized information useful [27].

### Previous Research on Breast Cancer Apps

Although studies have explored the integration of theoretical constructs in breast cancer interventions, little is known about theoretical underpinnings of breast cancer apps available to the general public. Despite the importance of the issue and the availability of the taxonomy that has been used effectively in other contexts [12,28,29], past analyses of cancer apps, in general, have focused primarily on the examination of content and functionalities of apps, without considering the importance of health behavior change techniques in app design. An analysis of the content of cancer apps found that the goal of cancer apps was mainly to raise awareness and to provide information, forfeiting opportunities to promote behavior change [18]. Only 1 study explored the integration of behavior change techniques into cancer-related apps, reporting missed opportunities to take advantage of interactive and user-centered features that may support behavior change, such as personalization, review of goals, and feedback [29]. However, the study examined apps for cancer survivors only, defined as individuals “diagnosed with cancer from the time of diagnosis through the balance of life” [29]; thus, this prior study did not take into account cancer apps developed for prevention purposes. Furthermore, data collection took place in 2013, representing an early stage in the development and dissemination of cancer apps. Since 2013, the number of health apps downloaded from major app stores has more than doubled, stressing the need for further evaluation [30].

Only 3 previous studies analyzed breast cancer apps specifically, examining the prevalence of gamification elements [31], the degree of medical professional involvement [32], and adherence to health literacy and interactivity strategies [33]. Findings across these studies suggest a lack of involvement of health experts in app design and content as well as limited adherence to theory- and evidence-based constructs [31-33]. However, 2 of the studies analyzed apps based on their description on the app store [31,32], which may not accurately reflect the content provided in the apps. Moreover, although theoretical guidance is paramount and a recommended best practice [10,12], none of these studies examined the use of behavior change theories and constructs in app content and design across the cancer care continuum.

In view of the difficulties facing consumers in identifying quality apps, consumer ratings might be a helpful tool. The 2 measures currently employed by the major app stores that indicate the quality of apps are peer star ratings and written reviews from users [34]. However, previous studies identified major flaws in these rating systems, as only a minority of apps received written reviews or star ratings, and written reviews were found to be unstructured, subjective, and short [31,32]. This lack of standardized measures in app stores’ rating systems makes it difficult for users to find apps that are of high quality in terms of content and design [31,32].

Nevertheless, star ratings and written peer reviews are the only 2 measures available to users that indicate the quality of apps, and it would thus be beneficial to examine whether users’ ratings accurately reflect the quality of apps. Our past study identified a relationship between star ratings and adherence to literate design, with higher star ratings being positively correlated with higher health literacy scores [33]. Similarly, apps that integrated evidence- and theory-based constructs were found to receive higher ratings [34]. Although app users are probably unaware of these constructs, these findings may suggest that they intuitively prefer evidence- and theory-based app design. It is possible that the integration of evidence- and theory-based constructs makes apps more effective, which increases user satisfaction, and, in turn, leads to higher ratings. Identifying whether the integration of behavior change techniques into app design is related to star ratings could provide additional insight into the accuracy of star ratings to measure app quality. We thus hypothesized that breast cancer apps that incorporate behavior change techniques would receive higher star ratings.

### Study Aims

Breast cancer has specific prevention, treatment, and care management and survivorship information, behavior, and support needs, and therefore, studies should explore the availability, content, and quality of cancer-specific apps. Given that apps have shown success in supporting healthy behaviors along the cancer care continuum, the goal of this study was to focus on commercial breast cancer apps that target behavior change. In contrast to a previous study of breast cancer apps’ content and health literacy standards that we conducted in 2016 [33], this study sought to focus primarily on the use of behavior change techniques integrated into app design supporting both preventive and postdiagnosis behavior change. Specifically, we sought to answer the following research questions (RQs):

RQ1: How many breast cancer apps are available on the iOS and Android app stores that focus on behavior change across the cancer care continuum?

RQ2: To what extent do breast cancer apps that seek to support behavior change include content along the cancer care continuum?

RQ3: To what degree are breast cancer apps theory based?

Given that star ratings are one of the only publicly available measures to indicate the quality of apps and previous studies have found positive correlations between evidence- and theory-based constructs and star ratings, we constructed the following hypothesis:

H1: Star ratings of the quality of apps will be positively associated with the degree to which apps incorporate behavior change techniques.

## Methods

### Sampling

**Figure 1:**
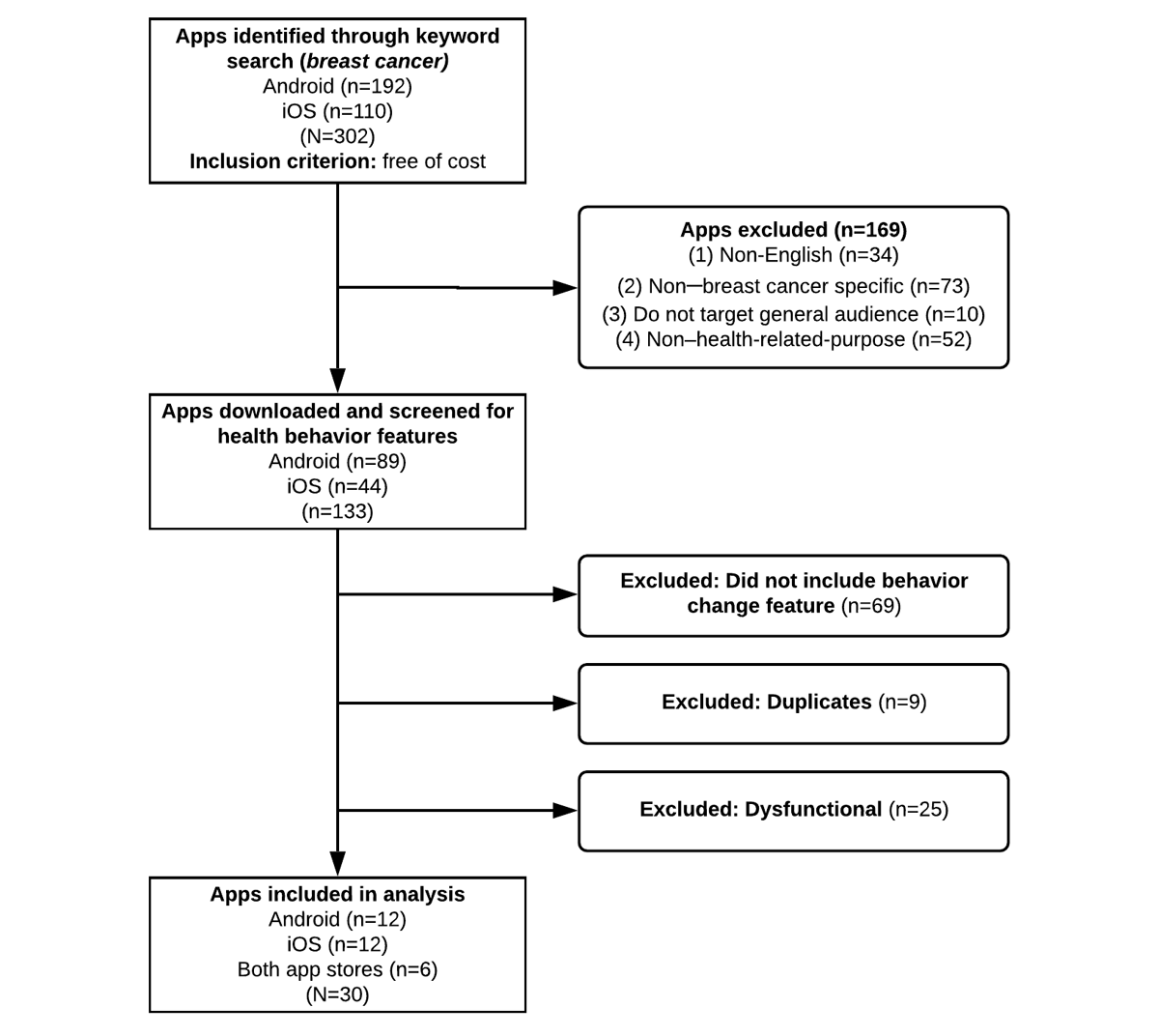
Screening process for sample selection.

The screening process and content analysis of breast cancer apps followed procedures outlined in previous content analyses of health apps [28,33]. In February 2018, the iOS App Store and the Android Play Store were screened for relevant apps using tablet devices. Only apps that were free of cost were included in the analysis, as previous studies suggest that users are reluctant to pay for apps and prefer apps that are free of cost [29,33]. The search term, *breast cancer*, was typed into the search bars in both app stores, and data on all free apps were transferred to a spreadsheet. A total of 302 free apps were identified in both app stores (192 for Android and 110 for iOS). Apps were included in the further analysis if they were (1) in English, (2) specific to breast cancer, (3) for the general public (as opposed to health professionals), and (4) developed for health promotion or prevention purposes (as opposed to providing screen savers or conference information). A total of 133 apps (89 Android and 44 for iOS) met the inclusion criteria and were downloaded onto tablet devices. The primary focus of this study was on the analysis of behavior change apps, and consequently, apps were screened for features or functions that aimed at supporting behavior change. Features or functions defined as supporting behavior change were reminders, scheduling options, interactive questionnaires, and similar features that clearly aimed at supporting certain behaviors, as opposed to only providing information. Several apps were duplicates (n=9) or did not open or crashed (n=25) and were excluded, leading to a final sample size of 30 breast cancer apps containing features that clearly aimed at supporting behavior change. See Figure 1 for the screening process and exclusion and inclusion of apps.

### Coding Process

The coding scheme used in this study was adapted from the previous analysis conducted in 2016 and included coding items related to the purpose of the app and evidence-based best practices along the cancer care continuum [33]. Assessment of behavior change theories followed a coding scheme based on Abraham and Michie’s [12] taxonomy of behavior change techniques and adapted to the coding of behavior change techniques in cancer apps [29].

A total of 2 graduate students were trained in three 2-hour-long sessions to ensure consistency in conceptualizations of coding items. A sample of 10 apps was coded to test for intercoder reliability. Intercoder reliability was calculated using Krippendorff alpha (see Table 1). Alphas for 26 variables were between .79 and 1, indicating excellent intercoder reliability. Alphas for the remaining 6 variables were not calculated because there was no data variance. Disagreements were discussed until consensus was reached.

**Table 1:** Krippendorff alpha values and percentage of agreement for each item.

Items		Krippendorff alpha	Percentage of agreement
**Content**			
	Primary prevention	1	100
	Genetic risk	1	100
	Genetic screening	1	100
	Mammography	1	100
	Clinical breast examination	1	100
	Self-breast examination	1	100
	Symptoms	1	100
	Stage	1	100
	Type of tumor	1	100
	Prognosis	1	100
	Treatment options	1	100
	Side effects	1	100
	Care management	1	100
	Prevention pills	1	100
	Survivorship	Undefined^a^	100
	End-of-life care/hospice	Undefined^a^	100
	Biological process	1	100
	Clinical trials	1	100
	Research referenced	1	100
**Behavior change techniques**			
	Customization	1	100
	Health-behavior link	1	100
	Behavior/consequences	.79	90
	Intention formation	1	100
	Goal setting	1	100
	Review of goals	Undefined^a^	100
	Instructions	Undefined^a^	100
	Materials/education	1	100
	Self-monitoring	Undefined^a^	100
	Persuasion	1	100
	Peer behavior	1	100
	Social comparison	1	100
	Mobilize social norms	Undefined^a^	100

^a^Krippendorff alpha is undefined when there is no expected disagreement between coders. This happens when all coders code a particular variable the same for every case, leading to division by zero in the calculation of alpha.

### Coding Scheme

A coding scheme was developed, which included information on app characteristics and user rating, the content of apps along the cancer care continuum, and the integration of behavior change techniques. The following sections provide an overview of how these categories were conceptualized and coded.

#### App Characteristics

General characteristics of each app were recorded, including the name of the app, developer, age rating, user star rating, and app category.

#### Primary Prevention

To assess apps’ content on prediagnosis behaviors, apps were analyzed for content related to preventive behaviors, such as being physically active and keeping a healthy weight.

#### Genetics

Apps were further coded for content related to (1) genetic risk and (2) genetic screening guidelines for individuals who may have an increased risk for developing breast cancer.

#### Early Detection

Information on early detection of breast cancer was coded for the following items: (1) mammography, (2) clinical breast examination, (3) self-breast examination, and (4) symptoms.

#### Diagnosis

Apps were coded for information on diagnosis of breast cancer, including content regarding (1) cancer stage, (2) tumor type, and (3) prognosis.

#### Breast Cancer Management and Therapeutics

Breast cancer apps were further analyzed for content related to breast cancer care and management following a breast cancer diagnosis, including the following items: (1) treatment options, (2) side effects, (3) care management and medication, and (4) prevention pills (eg, tamoxifen for individuals with increased risk).

#### Survivorship and End-of-Life Care

To assess whether apps provided information postdiagnosis and treatment, we coded for content related to survivorship and end-of-life care or hospice.

#### Research and Science

Apps were also coded for (1) containing biological information, such as explaining the biological process of developing breast cancer; (2) providing information on clinical trials; and (3) providing references for further biological and research-based information.

#### Behavior Change Techniques

The integration of behavior change techniques was assessed using a coding scheme previously developed to code behavior change techniques in cancer survivorship apps [29]. This coding scheme was based on Abraham and Michie’s [12] taxonomy of behavior change techniques but adapted to the analysis of apps developed for cancer survivors. Behavior change techniques included in the coding scheme were associated with several behavior change theories, including the Elaboration Likelihood Model [35], the social cognitive theory [36], the control theory [37], operant conditioning [38], the information-motivation-behavioral skills model [39], and the theory of planned behavior [40]. Further included were items related to tailored health communication and social support [41,42]. The final coding scheme included the following categories: (1) customization (ie, personalization or tailoring), (2) information/behavior relationship (ie, health-behavior link, behavior and consequences), (3) intention (ie, intention formation, goal setting, and review of goals), (4) facilitation (ie, instructions and information/education), (5) self-efficacy (ie, self-monitoring of goals and persuasion), and (6) social influence (information on peer behavior, comparison, and mobilizing social norms). These 6 categories contained a total of 13 specific behavior change techniques, which were summed to create a *behavior change technique score* with a possible range of 0 to 13, which was used for the statistical analysis to test H1.

### Statistical Analysis

SPSS version 25 (IBM Corp) was used to examine the relationship between the occurrence of behavior change techniques in apps and star ratings. Significance was determined at an alpha level of .05.

## Results

### Availability and Characteristics of Breast Cancer Apps Supporting Behavior Change

The first RQ sought to identify the availability of breast cancer apps that focused on supporting behavior change. The search resulted in a total of 302 free apps, of which 133 were identified as including breast cancer content. Only 30 apps met the inclusion criteria for supporting behavior change and were further analyzed. Multimedia Appendix 1 lists all 30 apps that were included in the final sample and provides characteristics and the behavior change technique score for each app.

Of the 30 apps analyzed, 12 (40%) were available on iOS only, 12 (40%) were available on Android only, and the others were available on both platforms (6/30, 20%). Most apps were rated either for everyone (12/30, 40%) or for users aged 12 years and older (10/30, 33%). There were also 3 apps rated for users aged 4 years and older, 3 (10%) rated for users aged 17 years and older, and 2 (6%) that did not have age ratings. The majority of the apps were categorized under either *medical* (13/30, 43%) or *health and fitness* (12/30, 40%).

### Apps’ Content Along the Cancer Care Continuum

The second RQ focused on the information presented in apps along the cancer care continuum. Table 2 provides a breakdown of the frequency of items coded in each stage of the cancer care continuum, ranging from primary prevention to survivorship or end-of-life care.

**Table 2:** Frequency of breast cancer app items (N=30).

Category and items		Value, n (%)	Behavior change technique score, mean (SD)
Primary prevention		13 (43)	5.31 (2.93)
**Genetics**			
	Risk	12 (40)	5.67 (2.46)
	Screening	5 (16)	6.40 (2.70)
**Early detection**			
	Mammography	12 (40)	5.25 (2.45)
	Clinical breast examination	10 (33)	5.10 (2.28)
	Self-breast examination	21 (70)	5.04 (2.58)
	Symptoms	21 (70)	5.38 (2.52)
**Diagnosis**			
	Stage	3 (10)	8.00 (1.73)
	Type of tumor	2 (6)	7.50 (2.12)
	Prognosis	3 (10)	7.33 (1.53)
**Management/therapeutics**			
	Treatment options	7 (23)	6.71 (2.50)
	Side effects	4 (13)	6.25 (2.50)
	Care management	6 (20)	6.33 (2.50)
	Prevention pills	3 (10)	8.33 (1.15)
**End-of-life care/hospice**			
	Survivorship	4 (13)	8.25 (0.96)
	End-of-life care/hospice	0 (0)	N/A^a^
**Research/science**			
	Biological process	4 (13)	7.00 (3.46)
	Clinical trials	3 (10)	7.00 (1.00)
	Research referenced	9 (30)	6.00 (2.94)

^a^N/A: not applicable.

With regard to prediagnosis behaviors and information, fewer than half of all apps addressed primary prevention (13/30, 43%), dealt with genetic risk (12/30, 40%), or provided recommendations for screening for genetic risk (5/30, 16%). Most apps offered guidance about self-breast examinations (21/30, 70%) and the symptoms of breast cancer (21/30, 70%). Fewer than half of the apps included information about mammography (12/30, 40%) or clinical breast examinations (10/30, 33%). With regard to diagnosis, 3 (10%) apps included information on cancer stage or prognosis, and 2 (6%) apps addressed the type of tumor. Similarly, content related to breast cancer management and therapeutics was discussed in less than one-fourth of all apps. More specifically, treatment options were discussed in 7 (23%) apps, care management in 6 (20%) apps, side effects in 4 (13%) apps, and prevention pills in 3 (10%) apps. Only 4 (13%) apps addressed survivorship, and there was no app that included information about end-of-life care.

**Figure 2:**
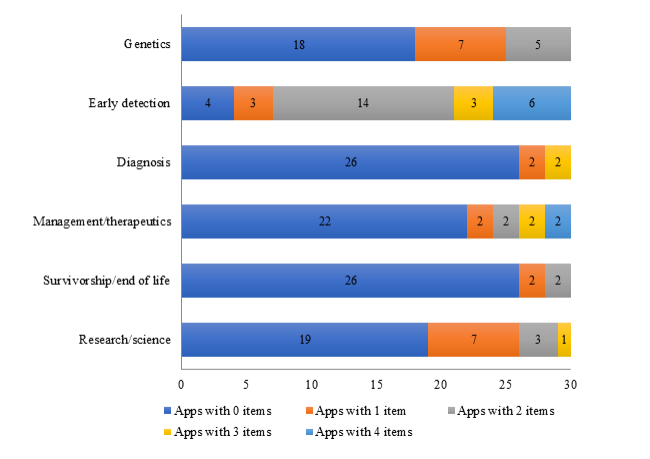
Comprehensiveness of apps’ (N=30) content on each cancer care continuum stage.

Figure 2 shows the comprehensiveness in which apps addressed each stage in the cancer care continuum by reporting on the number of relevant items they addressed. As explained earlier, each stage of the cancer care continuum was coded with several items, with the exception of primary prevention (refer to Table 2 for a breakdown of items coded in each stage of the cancer care continuum). For example, content related to genetics was coded with 2 items, namely (1) genetic risk and (2) genetic screening. Specifically, 18 (60%) apps did not address either 1 of the 2 items related to genetics, 7 (23%) apps addressed 1 item, and 5 (16%) apps addressed both of the items. Early detection received the most detailed attention. In addition, 3 (10%) apps contained 1 item related to early detection, 14 (46%) apps contained 2 items from this cancer stage, 3 (10%) apps contained 3 items, and 6 (20%) apps contained all 4 items. Only 4 (13%) apps did not address any of the items.

Diagnosis-related items, which included cancer stage, tumor type, and prognosis, were absent from most apps (26/30, 86%). Moreover, 2 (6%) apps addressed all 3 items, and 2 (6%) apps discussed only 1 of the 3 items. Similarly, the majority of apps did not discuss items related to breast cancer management (22/30, 73%), with only 8 (26%) apps including at least one item. Information related to survivorship and end-of-life care was also largely absent in apps (26/30, 86%), with 2 (6%) apps addressing 1 item and 2 (6%) apps addressing both items.

In addition to content related to each stage of the cancer care continuum, apps were also examined for the provision of scientific information, including the biological process of developing breast cancer, clinical trials, and citations. The majority of apps (19/30, 63%) included none of the 3 items, and 11 (36%) apps addressed at least one of the items.

### Use of Behavior Change Techniques in App Design

The third RQ focused on apps’ integration of behavior change techniques. As shown in Table 3, the most common element was related to facilitation. Most apps (24/30, 80%) provided instructions, although only 10 (33%) apps provided educational information on a specific health behavior. Most apps also contributed to knowledge and awareness by addressing the health-behavior link (21/30, 70%) and behavior and consequences (18/30, 60%). Nearly two-thirds of the apps allowed users to customize their experience through personalization and/or tailoring (19/30, 63%). Half of the apps (15/30, 50%) sought to prompt users to form intentions. No other behavior change technique appeared in more than one-third of the apps.

The possible range of the behavior change technique score was 0 to 13, although the actual range was 0 to 9 (mean 10 [SD 2.48])*.* Owing to the small sample, the assumptions of most parametric tests were not met, so such tests were not conducted. However, a behavior change technique score was calculated for each of the items (see Multimedia Appendix 1 for the behavior change technique score for each app). These numbers simply show the average number of behavior change techniques in apps with certain types of content. For example, the 3 apps dealing with chemoprevention pills had the highest average number of behavior change techniques (mean 8.33 [SD 1.15]), followed by the 4 apps addressing survivorship (mean 8.25 [SD 0.96]). The apps with the lowest average behavior change technique score were the 21 apps that dealt with breast self-examinations (mean 5.04 [SD 2.58]).

**Table 3:** Frequency of behavior change techniques (N=30).

Category and behavior change technique		Value, n (%)
**Customization**		
	Tailoring/personalization	19 (63)
**Information/behavior relationship**		
	Health-behavior link	21 (70)
	Behavior and consequences	18 (60)
**Intention**		
	Prompt intention formation	15 (50)
	Prompt goal setting	9 (30)
	Review of goals	7 (23)
**Facilitation**		
	Provides instructions	24 (80)
	Provides materials/education	10 (33)
**Self-efficacy**		
	Self-monitoring of goals	9 (30)
	Persuasion	8 (26)
**Social influence**		
	Information on peer behavior	7 (23)
	Social comparison (peer active)	6 (20)
	Mobilize social norms	0 (0)

### Relationship Between App Ratings and Prevalence of Behavior Change Techniques

We hypothesized that star ratings of apps and the integration of behavior change techniques would be positively associated. However, the small dataset made it impossible to directly test this hypothesis. Only half of the apps in our sample (n=15) had star ratings. The data were not normally distributed, and 2 apps accounted for a majority (n=593) of the total number of star ratings for all 15 apps (N=875). The median number of ratings per app was 20. The average star rating for these 15 breast cancer apps was quite high—4.3 on a 5-point scale (SD 0.67). No app that was rated earned an average lower than 3 stars.

Given the small number of available apps, it was not surprising that the Spearman rho test was not statistically significant. We decided to compare the number of behavior change techniques in apps that received star ratings and apps that received no star ratings. We theorized that star ratings are evidence that users are, at least somewhat, engaging with an app, and more behavior change techniques might make apps more engaging. As the data were not normally distributed, we used the Mann-Whitney *U* test to compare the 15 apps with user ratings against the 15 apps without user ratings. Apps with star ratings contained significantly more behavior change techniques (median 6.00) than apps without any star ratings (median 4.00; *U*=161.50; *z*=2.07; *P*=.04; *r*=0.38).

## Discussion

### Principal Findings

The goal of this study was to assess breast cancer apps currently available to users on the Android Play Store and iOS App Store for their inclusion of content along the cancer care continuum and their integration of behavior change techniques. Consistent with previous research, this study revealed significant shortcomings in apps’ adherence to evidence-based best practices and use of behavior change techniques [29,32].

The screening process of the Android and iOS app stores identified 133 breast cancer apps available to users, of which less than one-fourth had the clear goal of promoting healthy behaviors or facilitating behavior change through specific features or functions. This finding is consistent with previous findings [31,32] and reveals that most breast cancer app developers continue to focus on educational and informational purposes only. Although apps are important platforms for the dissemination of breast health information and resources, research suggests that providing information in itself is insufficient to promote behavioral changes [20]. These findings are particularly disappointing, given the advantage of mHealth in providing customized and interactive features that may enhance accessibility to health information and support behavior change.

Another concern relates to the prevalence of apps supporting breast self-examinations, which is a screening modality not recommended by leading health care organizations in the United States [15]. Research suggests that breast self-examinations are largely ineffective at reducing mortality rates and may lead to adverse health outcomes because of increased invasive diagnostic procedures [43]. Perhaps not surprisingly, the 21 apps focusing primarily on enhancing breast self-examinations were also the apps with the lowest average behavior change technique score, indicating that design of the apps developed for primary prevention purposes likely lacks involvement of health professionals and behavioral scientists. These findings indicate the persistence of the trend identified in previous analyses of breast cancer apps criticizing a lack of theory and evidence-based concepts in app development [32,33]. Given the potential influence of apps on preventive behaviors, it is essential to ensure that app content follows guidelines that are evidence based and not likely to cause unnecessary harm to individuals. It is also noteworthy that although almost half of the apps provided information on genetic risk, only one-sixth included information on screening or testing for genetic risk. Individuals who have an increased risk of developing breast cancer are recommended to get screened more regularly and at an earlier age and providing at-risk women with these screening guidelines may increase their attendance of medical checkups and screenings [15].

In contrast to the number of apps promoting preventive behaviors, only a few apps focused on postdiagnosis behaviors, such as treatment and care management, survivorship, or end-of-life care. These findings are consistent with results from our earlier analysis in 2016 [33] but deviate from other content analyses of breast cancer apps. For example, a content analysis conducted in 2016 reported that few apps addressed prevention and early detection, whereas more apps focused on disease and treatment information and disease management [31]. Apps in the study were classified based on the app store description, which could explain the different findings. This analysis and the analysis done in 2016 [33] revealed major discrepancies between the description of apps on the app store and their actual content. Hence, our findings underline the importance of downloading and analyzing apps, which is a rigorous and time-intensive approach to analysis compared with relying on apps’ content descriptions on the app stores.

Given the potential of apps to increase healthy behaviors in breast cancer patients, the less number of apps targeting postdiagnosis behaviors reveals missed opportunities to support care management and treatment decisions [33]. In particular, with only 3 apps providing information on survivorship and no app focusing on end-of-life care, our findings emphasize the pressing need for apps providing information and support for posttreatment health promotion and medical management. For the growing number of breast cancer survivors, who experience specific care management support and needs, these findings are particularly disappointing [6].

Our findings further lend support to the need for increased attention to the integration of behavior change techniques into app content and design. Research strongly supports the use of behavior change theories in mHealth interventions; however, adherence to behavior change techniques varied widely across apps. Apps demonstrated strong results in the presentation of a health-behavior link, the discussion of consequences of health behaviors, and the provision of clear instructions to facilitate behavior change. Furthermore, customization options offered by the majority of apps are promising. Apps allowed users to customize content in different ways, including through the collection of personal data, tailoring of content to specific diagnoses, or personal settings of reminder and scheduling functions. Our finding that most apps take advantage of interactive features to increase user engagement shows that apps have started to move away from a rather linear to a more user-centered presentation of content.

Although apps took advantage of the technical capabilities of the mobile devices to adapt content to users, they did not do so to increase self-efficacy in users or to encourage goal setting behaviors. This was particularly surprising considering the high number of features used in apps that sought to enhance these behavioral constructs. Examples of these features found in breast cancer apps included scheduling and reminder functions of mammogram appointments or medical checkups, journals to track changes in the breast, and diaries to record conversations with physicians or to note distress. Goal setting and self-monitoring have shown to effectively enhance behavioral changes, but the majority of apps fell short on introducing goal setting instructions that are actionable, providing an aggregated report of achieved goals, or prompting users to monitor their goals. Offering features without providing clear instructions and feedback may decrease the effectiveness of these functions to support behavior change. Similar results have been reported in earlier studies that identified the low scores of human-computer interaction components, such as control, tracking, and feedback systems [18,29]. Our findings, therefore, point at a disappointing lack of progress in the design of theoretically based cancer-related apps and in the integration of best practices.

Similarly, apps did not take advantage of social networking options to strengthen users’ social support system. Only 7 apps provided stories or experiences of other breast cancer patients or survivors, and only 6 apps allowed users to share their own story or to connect with others. None of the apps sought to mobilize social norms by exposing users to opinions from known or popular individuals. Apps are increasingly used as a communication and educational tool, and the sparse opportunities offered in breast cancer apps for individuals to connect with others undergoing similar experiences largely limit their potential to provide additional support. The lack of social media options and social influence has also been reported in analyses of cancer survivorship apps [29] and general cancer apps [18]. Given that social ties can increase patients’ well-being and the potential of apps to connect people across spatial boundaries, these findings are particularly disappointing.

Finally, our analysis highlights the need for safeguards and measures to increase the quality of available apps as has been identified in many previous analyses of health-related apps, including breast cancer apps [31,32]. Less than one-third of the apps reviewed in this study included references, whereas the majority of apps did not indicate their sources of information. This is concerning because references can help users evaluate the quality of information provided in apps.

In both the Android and iOS app stores, it is difficult for users to accurately assess the quality of health apps. The user rating system may serve as an indicator for app quality, but it does not necessarily reflect the reliability of information. As only half of all apps had user ratings, many apps do not provide users with any feedback regarding the quality of apps as assessed by users. The limitations of the data did not allow us to accurately test whether star ratings were correlated to the incorporations of behavior change techniques in app design. However, we did find that rated apps included more behavior change techniques than apps that were not rated. This finding is consistent with our previous study that reported a correlation between user rating and literate design [33]. Future research should continue to examine this relationship and whether apps that incorporate more behavior change techniques are more engaging for users. Moreover, as limitations in app stores’ rating systems impede identification of reliable and credible apps, there is a need to improve rating systems available to users.

### Strengths and Limitations of This Study

This is the first study focusing on the degree to which breast cancer apps that aim at initiating and supporting behavior change use constructs and features based on behavior change theories across the cancer care continuum. Although previous studies examined the overall content of breast cancer apps, including their adherence to health literacy principles [33], use of gamification elements [31], and purpose of breast cancer apps [31,32], this study is the first to primarily focus on apps that target behavior change.

There are also certain limitations to this study. First, this review only included free and commercially available breast cancer apps. It could be possible that apps developed by health care professionals or health care organizations are recommended to patients that are based on behavior change theories, but those were not included in this analysis. Second, we only focused on apps that were specifically created for breast cancer; as a result, we may have missed apps that included breast cancer information in general cancer apps. Third, this analysis reviewed apps in English only, and we may have missed apps developed for specific cultural groups. Finally, we used a shortened version of the taxonomy of behavior change techniques as a coding scheme. Other frameworks are available that could further guide the analysis of behavioral constructs in app design, for example, from a motivational or goal-oriented perspective [44]. Similarly, we did not include app-level data that would indicate actual app usage, which could provide insight into how users use breast cancer apps.

### Conclusions

Mobile apps have the potential to reduce health disparities and to overcome barriers of limited access to health care services and resources by providing evidence- and theory-based interventions to individuals. In view of the global breast cancer burden, mHealth interventions can serve as promising platforms to enhance preventive and postdiagnosis behavior change. However, our analysis shows that current breast cancer apps are disproportionally focused on behaviors to enhance primary prevention and early detection, most of which are not evidence based, whereas only few apps support care management, treatment, and posttreatment behaviors. Moreover, the analysis of behavior change techniques used in apps revealed significant shortcomings in apps’ use of goal setting and social influence features, which may decrease effectiveness in improving the overall health and well-being of individuals. More attention needs to be paid to the involvement of health professionals and behavioral and communication scientists in app development and adherence to theories in the design of mHealth apps to provide individuals with the support they need along the cancer care continuum.

## Appendix

Multimedia Appendix 1 List of breast cancer apps in final content analysis ranked by behavior change technique score (N=30).

## Abbreviations

mHealth: mobile health
RQ: research question
