# Supplementary material for: Use of Evidence-Based Best Practices and Behavior Change Techniques in Breast Cancer Apps: Systematic Analysis
Source: JMIR Mhealth Uhealth. 2020 Jan 24;8(1):e14082. doi: 10.2196/14082 (PMC7007595; doi:10.2196/14082)
Supplement: Multimedia Appendix 1 [file mhealth_v8i1e14082_app1.pdf]

Multimedia Appendix 1. List of breast cancer apps in final content analysis ranked by behavior change technique score (N=30).

App name	Developer	Platform	Category	Age rating	Star rating, mean	Total ratings	Behavior change technique score (0-13)
B4BC: Boarding for Breast Cancer	Black Sun Productions, Inc.	iOS/Android	Lifestyle	4+	5	6	9
Breast cancer	Anastore	Android	Medical	Everyone	3.4	9	9
Breast Cancer App	HealthCare Global Enterprises Ltd	Android	Medical	Unrated	4.8	21	9
Managing Breast Cancer with My Physician	@Point of Care	iOS	Medical	12+	N/A[a]	0	9
Breast Check Now	Breast Cancer Now	Android	Health and fitness	Everyone	4.1	33	8
Know: BRCA	ORAU	iOS/Android	Medical	Unrated	5	3	8

Michelle's Place	Michelle's Place Breast Cancer Resource Center	iOS	Business	12+	N/A	0	8
Your Man Reminder	Rethink Breast Cancer	Android	Health and fitness	Everyone	4.4	20	8
Stay on Course	Roche	Android	Medical	Everyone	3	1	7
Breast Self-Exam	Webfoot Technologies Inc	iOS	Health and fitness	12+	4.5	11	6
Breast Test	Positiva	Android	Health and fitness	Everyone	4	144	6
My Cancer Coach	Genomic Health Inc	Android	Medical	Everyone	4.4	25	6
Check Yourself!	Keep A Breast Foundation	iOS/Android	Lifestyle	17+	N/A	0	5
Altus Women's Center	Altus Women's Center	iOS	Medical	12+	N/A	0	4
Breast and Pec Check	YAP	iOS/Android	Health and fitness	4+	N/A	0	4
Mamocare	Sonix Apps	Android	Health and fitness	Everyone	4.9	10	4
Pink Knights	Zulekha Hospital LLC	iOS	Health and fitness	12+	N/A	0	4

Breast Friends App	Barry O'Mahony	iOS	Medical	12+	N/A	0	4
Komen: Connected for the Cure	T2 Studios	iOS	Medical	12+	N/A	0	4
Don't Forget to Check	Brawer Software	iOS	Health and fitness	17+	N/A	0	4
Najah Breast Cancer Apps	ASM Technologies	Android	Medical	Everyone	3	4	4
NCP Period Tracker	Alaa Haidar	iOS	Medical	12+	N/A	0	4
ASPH Breast Care	Mark Hinchcliffe	iOS	Health and fitness	17+	N/A	0	3
Check Mate	Bagher Bachcha Digital	Android	Health and fitness	Everyone	4.7	50	3
Breast Aware BCI	iPLANiT Apps	iOS/Android	Health and fitness	Everyone	N/A	0	3

Breast Self-Exams	Joaquin L. Garcia Moreno	Android	Medical	Everyone	4	89	3
Squeeze the Day	Dyn4m0 Consulting AB	iOS	Health and fitness	12+	N/A	0	3
Breast Cancer	Codore	iOS/Android	Medical	4+	N/A	0	2
Modo Rosa	1tucan: Super-Intelligent Kids	Android	Communication	Everyone	4.7	449	2
iCheck	Julie Sparkle	iOS	Education	12+	N/A	0	0

[a]N/A: not applicable.
